# Pathogenic Bi-allelic Mutations in *NDUFAF8* Cause Leigh Syndrome with an Isolated Complex I Deficiency

**DOI:** 10.1016/j.ajhg.2019.12.001

**Published:** 2019-12-19

**Authors:** Charlotte L. Alston, Mike T. Veling, Juliana Heidler, Lucie S. Taylor, Joseph T. Alaimo, Andrew Y. Sung, Langping He, Sila Hopton, Alexander Broomfield, Julija Pavaine, Jullianne Diaz, Eyby Leon, Philipp Wolf, Robert McFarland, Holger Prokisch, Saskia B. Wortmann, Penelope E. Bonnen, Ilka Wittig, David J. Pagliarini, Robert W. Taylor

**Affiliations:** 1 Wellcome Centre for Mitochondrial Research, Newcastle University, Framlington Place, Newcastle upon Tyne, NE2 4HH, UK; 2 NHS Highly Specialised Services for Rare Mitochondrial Disorders, Royal Victoria Infirmary, Newcastle upon Tyne Hospitals NHS Foundation Trust, Queen Victoria Road, Newcastle upon Tyne, NE1 4LP, UK; 3 Morgridge Institute for Research, Madison, WI 53715, USA; 4 Department of Biochemistry, University of Wisconsin—Madison, Madison, WI 53706, USA; 5 Department of Systems Biology, Harvard Medical School, Boston, MA 02115, USA; 6 Functional Proteomics, Goethe-Universität, Frankfurt am Main, 60590 Frankfurt, Germany; 7 Department of Molecular and Human Genetics, Baylor College of Medicine, Houston, TX 77030, USA; 8 Manchester Centre for Genomic Medicine, Saint Mary’s Hospital, Oxford Road, Manchester, M13 9WL, UK; 9 Academic Unit of Paediatric Radiology, Royal Manchester Children’s Hospital, Manchester University Hospitals NHS Foundation Trust, Oxford Road, Manchester, M13 9WL, UK; 10 Division of Informatics, Imaging, and Data Sciences, School of Health Sciences, Faculty of Biology, Medicine, and Health, University of Manchester, Oxford Road, Manchester, M13 9PT, UK; 11 Rare Disease Institute, Children’s National Hospital, Washington, DC 20010, USA; 12 DRK Kinderklinik, Siegen, Wellersbergstraße 60, 57072 Siegen, Germany; 13 Institute of Human Genetics, Technische Universität München, 81675 München, Germany; 14 Institute of Human Genetics, Helmholtz Zentrum München, 85764 Neuherberg, Germany; 15 Department of Pediatrics, Salzburger Landeskliniken (SALK), Paracelsus Medical University (PMU), 5020 Salzburg, Austria; 16 German Center for Cardiovascular Research (DZHK), Partner Site RheinMain, 60590 Frankfurt, Germany

**Keywords:** mitochondrial disease, complex I deficiency, NDUFAF8, molecular diagnosis

## Abstract

Leigh syndrome is one of the most common neurological phenotypes observed in pediatric mitochondrial disease presentations. It is characterized by symmetrical lesions found on neuroimaging in the basal ganglia, thalamus, and brainstem and by a loss of motor skills and delayed developmental milestones. Genetic diagnosis of Leigh syndrome is complicated on account of the vast genetic heterogeneity with >75 candidate disease-associated genes having been reported to date. Candidate genes are still emerging, being identified when “omics” tools (genomics, proteomics, and transcriptomics) are applied to manipulated cell lines and cohorts of clinically characterized individuals who lack a genetic diagnosis. NDUFAF8 is one such protein; it has been found to interact with the well-characterized complex I (CI) assembly factor NDUFAF5 in a large-scale protein-protein interaction screen. Diagnostic next-generation sequencing has identified three unrelated pediatric subjects, each with a clinical diagnosis of Leigh syndrome, who harbor bi-allelic pathogenic variants in *NDUFAF8*. These variants include a recurrent splicing variant that was initially overlooked due to its deep-intronic location. Subject fibroblasts were found to express a complex I deficiency, and lentiviral transduction with wild-type *NDUFAF8*-cDNA ameliorated both the assembly defect and the biochemical deficiency. Complexome profiling of subject fibroblasts demonstrated a complex I assembly defect, and the stalled assembly intermediates corroborate the role of NDUFAF8 in early complex I assembly. This report serves to expand the genetic heterogeneity associated with Leigh syndrome and to validate the clinical utility of orphan protein characterization. We also highlight the importance of evaluating intronic sequence when a single, definitively pathogenic variant is identified during diagnostic testing.

## Main Text

Leigh syndrome (MIM: 256000) is one of the most common neurological phenotypes observed in pediatric mitochondrial disease presentations, with an estimated incidence of one per 40,000 births.^1^ Clinical diagnosis is supported by characteristic neuroimaging findings, with symmetrical lesions in the basal ganglia, thalamus, and brainstem, and is associated with a loss of acquired cognitive, visual, and motor skills. Onset is typically in infancy or early childhood, though adult-onset Leigh syndrome is reported.^2^^,^^3^ Although it is phenotypically well defined, its vast genetic heterogeneity, with >75 disease-associated genes having been reported to date, makes identification of the genetic defect challenging.^4^ Causative pathogenic variants have been identified in both the nuclear genome and the mitochondrial genome (mtDNA), and these variants affect various aspects of mitochondrial function including structural subunits and assembly factors of respiratory chain complexes (e.g., *NDUFS2* [MIM: 602985]^5^ and *SURF1* [MIM: 185620]^6^), Krebs cycle components (e.g., *PDHA1* [MIM: 300502]^7^), mitochondrial protein translation (e.g., *MTFMT* [MIM 611766]^8^), and valine metabolism (e.g., *ECHS1* [MIM 602292]^9^).

Evolving from the 100,000 Genomes Project in the UK, the newly established National Health Service (NHS) Genomic Medicine Service promises to revolutionize the genetic diagnosis of heterogeneous conditions such as Leigh syndrome through clinical whole-genome sequencing,^10^ with similar programs occurring elsewhere including the Bavarian Genome Project in Germany and at the Victorian Clinical Genetics Services (VCGS) in Melbourne, Australia. For many affected individuals though, the success of the NHS Genomic Medicine Service relies on the identification and characterization of novel candidate genes in the research setting. The application of emerging “omics” tools—including but not limited to genomics, proteomics, and transcriptomics—to cohorts of clinically characterized individuals who lack a genetic diagnosis continues to identify novel candidate genes for Leigh syndrome, including *TIMMDC1* (MIM: 615534), which was found through the use of transcriptomics,^11^ and *NDUFAF8* (MIM: 618461), which was found through mass spectrometry-based proteomic analyses.^12^ NDUFAF8, previously known as the orphan protein C17orf89, was highlighted as a potential complex I assembly factor based on its strong protein-protein interaction with the well-characterized complex I assembly factor NDUFAF5 (MIM: 612360).^12^ RNAi knockdown of *NDUFAF8* expression in HEK293 cells caused a dramatic reduction in both NDUFAF5 protein levels and complex I activity, providing further compelling evidence of an interaction.^12^ We previously identified a severe reduction in *NDUFAF8* (*C17orf89*) expression in an unresolved case of complex I deficiency,^12^ but failed to identify any potential underlying pathogenic variants. Here, inclusion of this candidate gene in our molecular genetic diagnostic pipeline and collaboration with other diagnostic referral centers has led to the identification of three unrelated pediatric cases, each with a clinical diagnosis of Leigh syndrome and harboring bi-allelic variants in *NDUFAF8*. These variants included a recurrent splicing variant that was initially overlooked due to its deep-intronic location. This report serves to expand the genetic heterogeneity associated with Leigh syndrome and validates the clinical utility of orphan protein characterization; we also highlight the importance of evaluating intronic sequence when a single, definitively pathogenic variant is identified during diagnostic testing.

Subject 1 was the first child of non-consanguineous white, British parents, born at term after an uncomplicated pregnancy (birthweight 3.05Kg, ninth centile). He was initially breastfed and thought to be well until, at 3 months of age, he developed infantile spasms with a hypsarrythmic electroencephalogram (EEG) and was commenced on steroid (prednisolone) treatment. He was admitted at 4 months of age with a rhinoviral infection requiring optiflow respiratory support. Intussusception was suspected on account of his inconsolable crying, prompting transfer to the regional tertiary center. On arrival, he was found to have a metabolic acidosis with a maximal lactate of 15 mmol/L; intussusception was excluded and he developed apnoeic episodes requiring invasive ventilation.

**Figure 1:**
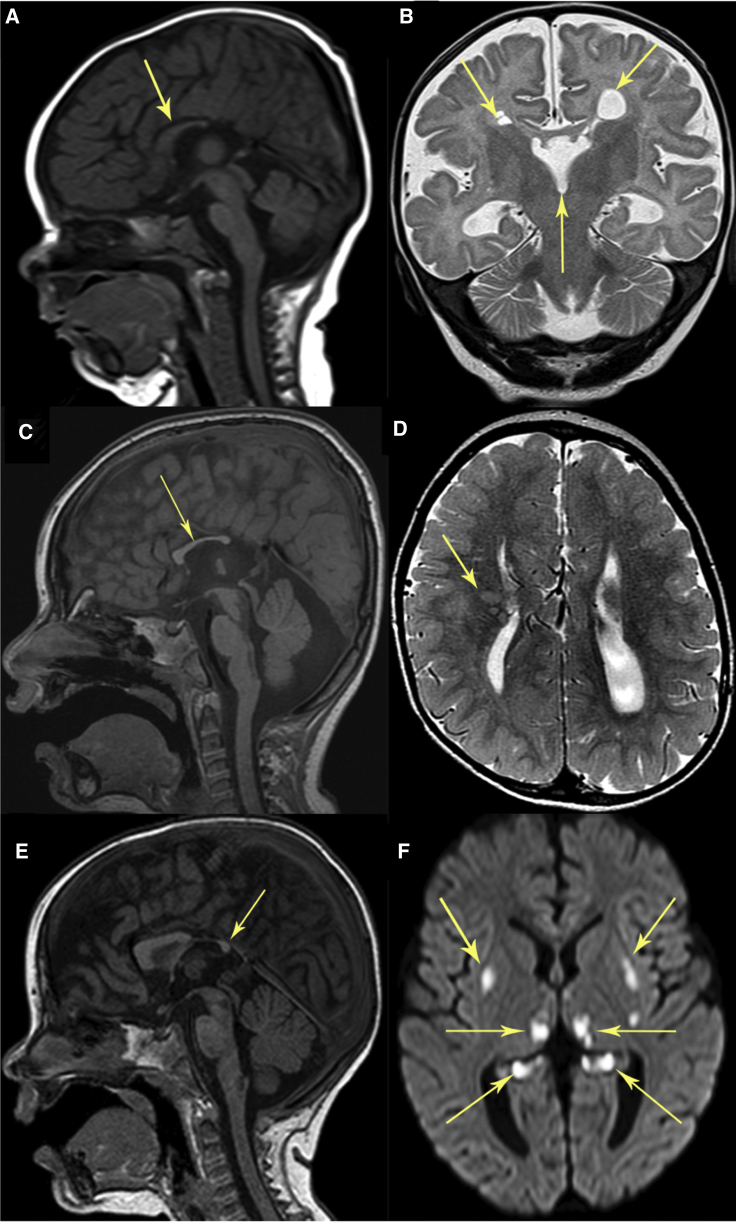
Neuroimaging of Subjects Harbouring Bi-Allelic Pathogenic *NDUFAF8* Variants (A and B) Subject 1 at 5 months of age. (C and D) Subject 2 at 2 years, 9 months of age. (E and F) Subject 3 at 1 year, 2 months of age. Sagittal T1-weighted imaging (T1WI) (A, C, and E) demonstrates dysmorphic corpus callosum (arrows): For subjects 1 and 2, no splenium is present (A and C), and only a small posterior part of the splenium is present in subject 3 (E). Sagittal T1WI (C and E) reveals signal abnormality in the dorsal brainstem corresponding to areas with restricted diffusion for subjects 2 and 3. Coronal T2-weighted imaging (B) of subject 1 demonstrates bilateral periventricular cysts and absent septum pellucidum (arrows). Axial T2-weighted imaging (D) depicts right frontal gray matter heterotopia in subject 2 (arrow). Axial diffusion-weighted imaging (F) shows bilateral symmetrical diffusion restriction in the putamina, thalami, and hippocampal tails in subject 3 (arrows).

Brain MRI (Figure 1) obtained at 5 months of age revealed bilateral symmetrical signal changes in the globi pallidi, thalami, brainstem, and optic radiations. In addition, there was evidence of bilateral frontal polymicrogyria and gray matter heterotopia (not shown), corpus callosal dysgenesis (Figure 1A), bilateral periventricular cysts, and absent septum pellucidum (Figure 1B). These MRI changes are consistent with a clinical diagnosis of Leigh syndrome, and involvement of the brainstem is likely to underlie his disrupted respiratory drive. Clinically, he was noted to have optic atrophy with pale discs and was generally hypotonic. He had elevated blood lactate (3.0–6.0 mmol/L; normal range, < 2 mmol/L) while urinary organic acids and acylcarnitine profiles were unremarkable. Subject 1 was discharged 2 weeks after admission; he remained generally hypotonic with increased tone in his lower limbs and was fed through a neurogastric (NG) tube. He has made steady albeit slow progress since; currently age 2 years, he is able to roll from front to back, can vocalise five to 10 words, is fully orally fed, and has visual impairment. He continues to have regular fleeting seizures but does not require regular anti-epileptic medication. His electrocardiogram (ECG) and echocardiogram were both normal, and he has good sustained growth on the 25^th^ centile for weight and height.

Subject 2 was born to unrelated white, American parents via caesarean section at 35 weeks following a pregnancy complicated by intrauterine growth restriction (birthweight 1.38 kg, <0.4^th^ centile). Prenatal ultrasound indicated prominent brain ventricles, possible left dysplastic kidney, and reversed end diastolic flow. A head ultrasound and brain MRI at birth, obtained due to severe intrauterine growth restriction (IUGR), showed large areas of bilateral periventricular cystic encephalomalacia with a thin corpus callosum and no acute hemorrhage. No abnormalities were noted in urine cytomegalovirus (CMV) and serum IgM levels. He spent 4 weeks in neonatal intensive care, receiving tube feeds, before discharge. He developed infantile spasms at 9 months of age, and an EEG revealed modified hypsarrhythmia. Small optic nerves and nystagmus were noted during ophthalmologic review. Delayed motor development was evident (rolling over at 6 months, sitting at 13–14 months, and pulled-to-stand at 15 months) at this time, but no regression was evident. By 2 years of age, he was still neither crawling nor standing independently, and he had no speech. A viral illness at 2 years, 9 months of age caused a developmental regression, although some skills were subsequently regained. Brain MRI (Figure 1) obtained at 2 years, 9 months of age demonstrated signal abnormality in the dorsal brainstem, corpus callosum dysgenesis (Figure 1C), right-sided gray matter heterotopia (Figure 1D), and bilateral periventricular cysts (not shown). He had a persistently elevated blood lactate (2.9–6.2mmol/L, ref < 2.0mmol/L) and an elevated lactate/pyruvate ratio (22, ref 10–20), but all other laboratory results were normal, including urinary organic acids, plasma organic acids, creatine kinase, serum MMA, ammonia, free/total carnitines, and IGF1. By age 3, his dysphagia had progressed so that feeding was taking an unfeasibly long time and, as a result, he began to lose weight. He was admitted age 3 years, 2 months for increasing lethargy and pneumonia, but he deteriorated further, developing respiratory failure that required intubation, tracheostomy, and a gastrostomy tube. Despite these efforts, subject 2 died at the age of 4 years, 1 month.

Subject 3 was the second child of healthy, non-consanguineous German parents; his older sister is healthy. His gestation, delivery, and neonatal phase were all unremarkable, and development was age-appropriate until the age of 15 months. He was mainly breastfed, and his parents reported he lacked drive to wean onto solid foods. At age 15 months, he presented with severe dehydration precipitated by two days of fever, vomiting, and diarrhea. At this time, his weight was 9.8 kg (30^th^ centile) and height was 84 cm (95^th^ centile). He was first admitted at 1 year, 2 months of age in hypertensive crisis; brain MRI (Figure 1) obtained at this time depicted bilateral symmetrical signal abnormality in the dorsal brainstem (Figure 1E), basal ganglia, thalamus, and hippocampi (Figure 1F) and corpus callosum dysgenesis (Figure 1E). Laboratory investigations reported metabolic acidosis (pH 7.24, pCO_2_ 28.2 mmHg, HCO_3_ 12.2 mmol/L, BE −13.8 mmol/L) with normal blood lactate 1.44 mmol/L (ref < 2mmol/L) and slightly elevated cerebrospinal fluid (CSF) lactate (3mmol/L, ref < 2.1mmol/L). Urinary organic acids demonstrated an increased concentration of Krebs cycle intermediates. He also had normochromic, normocytic anemia (Hb 7.9 mg/dl, MCV 80 fl, MCH 25.9 pg, MCHC 32.4 g/dl), folinic acid deficiency (1.4 ng/mL, ref > 12.2), and vitamin B12 deficiency (125 pg/mL, ref 400-883). One week after admission, he presented with inconsolable crying and in hypertensive crisis which was difficult to treat. He was discharged following gastrostomy (PEG) tube insertion on account of failure to thrive and feeding problems. He died during another hypertensive crisis at the age of 18 months.

**Figure 2:**
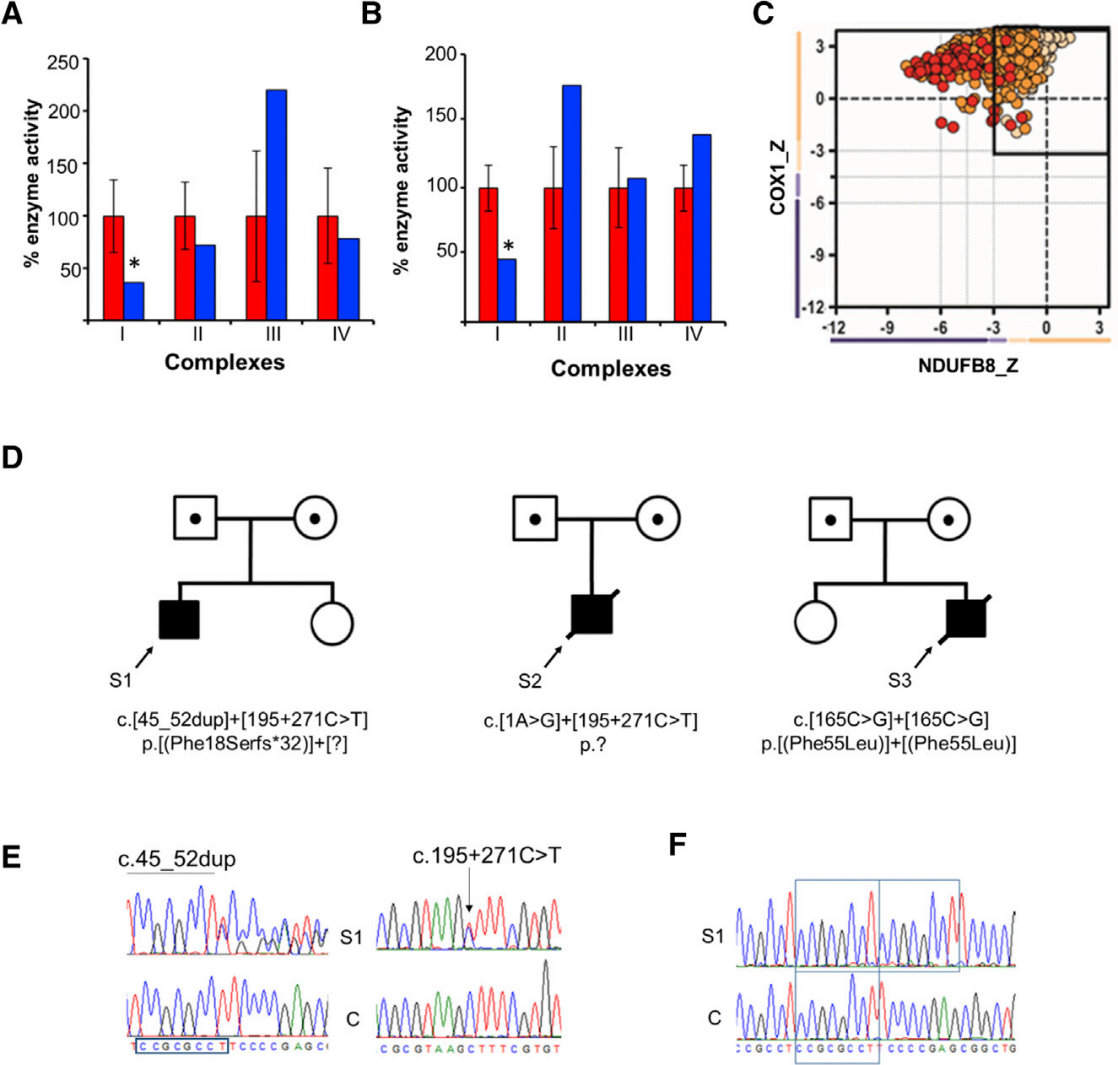
Bi-Allelic *NDUFAF8* Variants Are Identified in Three Unrelated Subjects Respiratory chain enzyme analysis reveals a marked isolated complex I deficiency in subject 1’s skeletal muscle biopsy (shaded blue) compared to controls (shaded red) (A); this finding is recapitulated in the fibroblasts from subject 1 (B). Mean enzyme activities of muscle controls (n = 25) and fibroblast controls (n = 10) are set to 100%, with error bars representing standard deviation. The asterisk denotes a significant loss of enzyme activity. (C) Quadruple immunofluorescent histochemical analysis of skeletal muscle biopsy from subject 1 demonstrates reduced levels of NDUFB8 (complex I) in the majority of single muscle fibers relative to those of the marker protein (porin), and expression of COX1 (complex IV) is preserved. Each dot represents a single muscle fiber. Black dashed lines represent the SD limits for the classification of the fibers. The X- and Y-axes represent the expression of NDUFB8 and COX-1: normal (< −1), intermediate +ve (−1 to −2 SD), intermediate −ve (−2 to −3 SD), and deficient (> −3 SD). The mean expression level of normal fibers is denoted a value of 0. Dots that fall outwith the solid box at -3 SD are strongly deficient fibres. Dots are color coded according to their mitochondrial mass (very low, blue; normal, beige; very high, red). (D) Family pedigrees of subjects 1, 2, and 3 and corresponding recessive *NDUFAF8* variants; compound heterozygous c.45_52dup (p.Phe18Serfs^∗^32) and c.195+271C>T (p.?) *NDUFAF8* variants in subject 1; compound heterozygous c.1A>G (p.?) and c.195+271C>T (p.?) *NDUFAF8* variants in subject 2 and a homozygous c.165C>G (p.Phe55Leu) *NDUFAF8* variant in subject 3. (E) Sequencing chromatograms depict the c.45_52dup and c.195+271C>T variants present in genomic DNA from S1. (F) cDNA studies using RNA derived from subject 1 fibroblasts show that only the c.45_52dup allele is present at the mRNA level, supporting the possibility of degradation of the transcript associated with the c.195+271C > T variant. S1 = subject 1; C = wild-type control. Variant nomenclature is according to GenBank accession NM_001086521.1

Informed consent for diagnostic and research studies was obtained for all subjects in accordance with the Declaration of Helsinki protocols and approved by local institutional review boards. Muscle biopsy was performed for subjects 1 and 3 due to clinical suspicion of a mitochondrial disorder. Routine histochemical investigations including sequential cytochrome *c* oxidase (COX)/succinate dehydrogenase (SDH) histochemistry and modified Gomori trichrome staining were unremarkable, with no COX-deficient fibers reported in either case. Spectrophotometric analysis of mitochondrial-enriched muscle homogenates (subjects 1 and 3) and skin fibroblasts (subject 1), were performed as previously described.^13^ Results supported a marked isolated complex I deficiency in the muscle (Figure 2A) and fibroblasts (Figure 2B) from subject 1, with complex I activity measured at 33% activity in muscle, and 47% in fibroblasts, relative to citrate synthase. Analysis of muscle biopsy from subject 3 revealed a residual complex I activity of just 3% (expressed relative to citrate synthase activity), although additional tissue was not available for further study. Quadruple immunofluorescent histochemistry^14^ supported an isolated complex I deficiency for subject 1 (Figure 2C). No muscle biopsy was available from subject 2 as a molecular diagnosis was sought at an early stage in the diagnostic pipeline. Molecular genetic investigations were initiated for all three subjects through the use of ethylenediaminetetraacetic acid (EDTA) blood DNA samples. A bespoke targeted strategy was employed for subject 1; in brief, a supplemental panel of short (200bp) amplicons corresponding to the coding exons (+/− 10bp at the intron boundaries) of *NDUFAF8* were spiked into an existing custom “complex I” ampliseq library for IonTorrent PGM sequencing. Library preparation, PGM sequencing, and bioinformatic analysis were performed essentially as reported.^15^ Whole-exome sequencing was performed for subjects 2 and 3 through the use of previously reported methodologies and interpreted according to American College of Medical Genetics (ACMG) guidelines.^11^^,^^16^^,^^17^ Analysis of the genetic data revealed candidate pathogenic variants in *NDUFAF8* (RefSeq: NM_001086521.1) for all three subjects (Figure 2D). Subjects 1 and 2 were each observed to have a single heterozygous class 5 ACMG variant, a c.45_52dup (p.Phe18Serfs^∗^32) duplication in subject 1 and a c.1A>G (p.?) variant in subject 2. Subject 3 was found to harbor an unreported homozygous c.165C>G (p.Phe55Leu) variant; amino acid Phe55 is invariant across Boreoeutheria to at least *Danio rerio,* so this finding supports potential functional importance (Figure S1). Given that the clinical presentations of subjects 1 and 2 were compatible with a complex I disorder, the presence of a single heterozygous pathogenic *NDUFAF8* variant in each subject prompted further investigation to determine whether additional undetected pathogenic variants were present in *NDUFAF8*.

The gene structure of *NDUFAF8* facilitated additional genetic investigations—the cDNA is just 225 base pairs across three exons that span ∼3 kb of genomic sequence (Figure S2). WES data for subject 2 included sufficient reads that mapped to the introns of *NDUFAF8* to enable detection of a heterozygous intronic variant, c.195+271C>T. In contrast, the sequencing strategy utilized for subject 1 resulted in minimal intronic sequence reads, necessitating further experimentation. A long-range PCR amplicon spanning the entire *NDUFAF8* locus was prepared for Ion Torrent PGM sequencing through the use of the Ion Xpress Plus Fragment Library Kit (Life Technologies) according to the manufacturer’s protocol. Analysis of the resultant sequence data revealed an identical heterozygous intronic c.195+271C>T variant in subject 1 (Figure 2E). The identified *NDUFAF8* variants reported here have been submitted to the ClinVar database (see Accession Numbers). To investigate whether the c.195+271C>T variant was associated with an mRNA defect, a fibroblast cell line from subject 1 was referred for cDNA sequence analysis. Whole RNA was extracted from subject and control fibroblasts and reverse transcribed as previously reported.^18^ Analysis was performed on confluent fibroblasts grown under either standard tissue culture conditions using Dulbecco modified eagle medium (DMEM) or with an additional overnight culture in emetine-containing media (100mg/mL) to preserve any mRNA transcripts that would normally be subject to nonsense-mediated mRNA decay.^19^ Sequencing analysis of PCR-amplified *NDUFAF8* transcripts supported the loss of the *NDUFAF8* allele that harbored the intronic c.195+271C>T variant, demonstrated by a loss of heterozygosity at the c.45_52 duplication locus (Figure 2F). PCR amplification and electrophoresis of the *NDUFAF8* transcripts through a 2% agarose gel facilitated identification of normal- and abnormal-length cDNA fragments (Figure S3). Amplicons from subject 1 and a healthy control were excised by crude band-stab, PCR-amplified, and subjected to Sanger sequencing; the results supported the likelihood of a loss of the functionally relevant transcript (isoform 2) in subject 1 fibroblast-derived cDNA, with only the non-functional isoform 3 transcript detectable.

**Figure 3:**
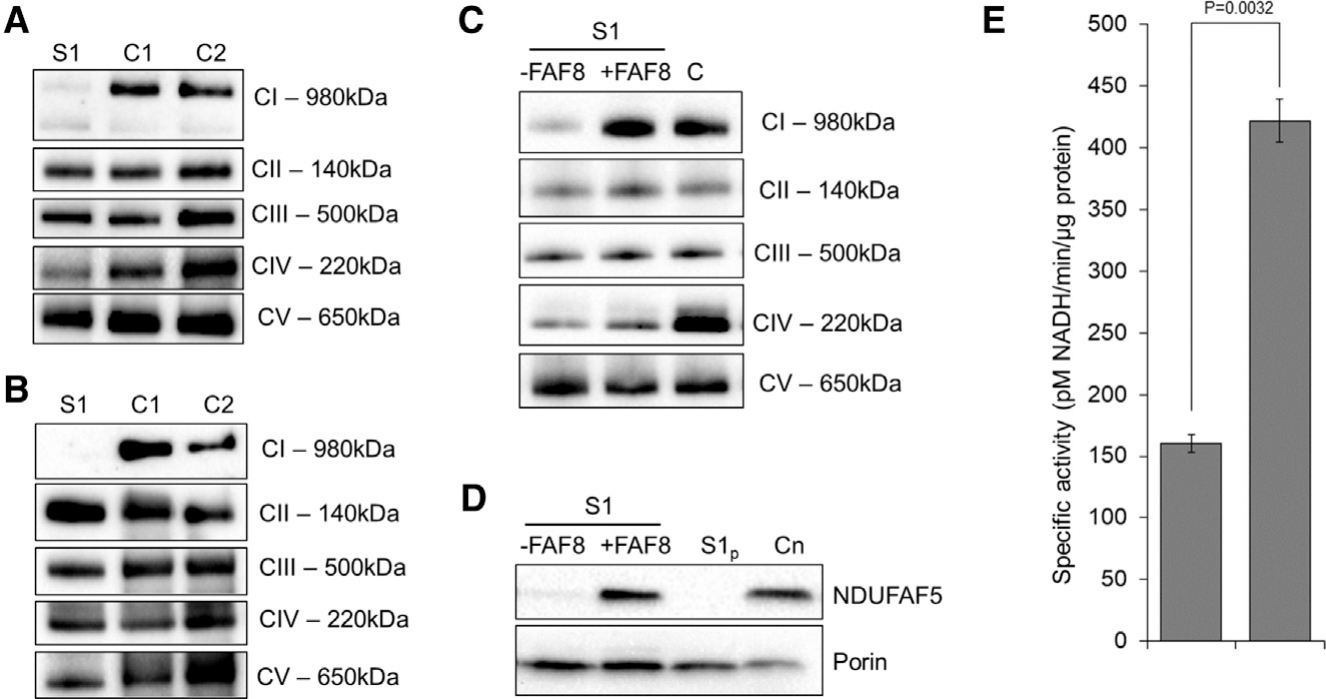
BN-PAGE and Complementation Studies (A and B) Mitochondria isolated from cultured skin fibroblasts (A) and skeletal muscle (B) from subject 1 and age-matched control subjects were solubilized in n-dodecyl β-d-maltoside (DDM) and subjected to BN-PAGE and immunoblotting analysis using antibodies directed to various OXPHOS complexes (complex I: NDUFB8; complex II: SDHA; complex III: Core2; complex IV: COX1; complex V: ATP5A). A striking reduction in assembled complex I was apparent in both the skeletal muscle biopsy and fibroblast cell line from subject 1 in comparison to controls. C, Wild-type *NDUFAF8* cDNA was generated and introduced into control and subject cell lines via lentiviral expression under the EF1α promoter. Enriched mitochondria were solubilized in DDM before BN-PAGE analysis. Immunoblotting using antibodies against the complex I subunit NDUFA9 (top) and the complex II subunit SDHA (bottom) as a loading control revealed less complex I in subject cell lines than in control cell lines. After transduction with *NDUFAF8* cDNA, complex I levels were restored. D, SDS-PAGE and western blotting of whole fibroblast cell lysates from subject 1 transduced with wild-type *NDUFAF8* cDNA (+FAF8) clearly demonstrates increased levels of NDUFAF5 relative to the empty vector (-FAF8) or untreated subject 1 fibroblasts (S1p), comparable to those observed in the healthy age matched control, C. E, Comparison of the complex 1 activity exhibited by subject 1 fibroblasts following transduction with either the empty vector (–FAF8) or wild-type *NDUFAF8* mRNA vector (+FAF8) cell lines corroborates functional rescue of the biochemical phenotype. Experimental data shown are derived from the results of three technical replicate assays; error bars are at 1 standard deviation. P value calculated as by Student’s t test.

Availability of muscle biopsy and a fibroblast cell line from subject 1 facilitated further characterization of his identified *NDUFAF8* variants. To investigate the oxidative capacity of his fibroblasts, high-resolution respirometry analysis of subject 1 and control fibroblasts was performed using the Oxygraph-2k platform and DatLab software v6.1.0.7, as described elsewhere.^20^ Respirometry demonstrates a reduction in oxidative capacity in the fibroblasts from subject 1 relative to controls (Figure S4). Blue-Native (BN)-PAGE of enriched mitochondrial fractions from muscle (Figure 3A) and fibroblasts (Figure 3B) of subject 1 and controls was performed as previously reported.^21^ Immunoblotting using antibodies raised against structural subunits from each OXPHOS complex (complex I [NDUFB8], complex II [SDH70], complex III [core 2], complex IV [COXI], and complex V [ATP5A]) revealed decreased levels of fully assembled complex I in both muscle cells and fibroblasts from subject 1 when compared to controls. All other OXPHOS complexes were unaffected, thus corroborating the hypothesis of involvement of subject 1’s *NDUFAF8* variants in his pathology.

In light of the marked complex I defect in the fibroblast cell line from subject 1, lentiviral rescue of the biochemical phenotype was attempted through reintroduction of a wild-type version of *NDUFAF8*. In brief, we generated a modified third-generation lentiviral construct that contained an EF1α promoter;^22^^,^^23^ the vector details are available on the Addgene database (see Web Resources). Cultured fibroblasts from subject 1 were infected with either a FLAG-tagged NDUFAF8-encoding lentivirus or an empty vector in order to generate two polyclonal cell lines: one line expressing *NDUFAF8-FLAG* (+AF8), and its empty vector counterpart (–AF8) that served as a negative control for off-target effects. NDUFAF8-FLAG production in the +AF8 cell line was confirmed by immunoblot (Figure S5). To determine whether a rescue was obtained following transduction with wild-type *NDUFAF8-FLAG* cDNA, BN-PAGE was performed on mitochondria-enriched fractions using the –AF8 and +AF8 lines (Figure 3C).

The results confirmed a substantial increase in fully assembled Complex I in the subject fibroblasts expressing *NDUFAF8-FLAG*, supporting the likelihood of a rescue of the complex I assembly defect for subject 1, with little effect on the assembly of the other complexes. In light of the previous association between NDUFAF5 and NDUFAF8, NDUFAF5 steady-state levels were assayed by SDS-PAGE of cell lysates (Figure 3D). Immunoblotting with an antibody raised against NDUFAF5 (Abcam ab192235) confirmed a loss of NDUFAF5 in subject 1’s primary cell line (S1p) and the subject line transduced with the empty vector (-AF8), a result consistent with previous investigations^12^ and supporting the likelihood of an association between NDUFAF5 and NDUFAF8 in mammalian cells. These data are further corroborated by the marked increase in the steady-state level of NDUFAF5 in subject 1’s NDUFAF8+ transduced line (+AF8). Porin (Abcam ab14734) was used as a loading control. Finally, spectrophotometric analysis of the +AF8 and -AF8 lines was undertaken, demonstrating a marked increase in complex I activity in subject 1’s NDUFAF8+ transduced line (+AF8); the empty vector (-AF8) cell line showed no measurable rescue when a colorimetric complex I activity assay (ab109721, Life Technologies) was used (Figure 3E). Data using the colorimetric assay (ab109721, Life Technologies), which demonstrates the complex I deficiency in subject 1 fibroblasts, are shown in Figure S6. Together, these data demonstrate that overexpression of *NDUFAF8* is sufficient to rescue complex I assembly, NDUFAF5 levels, and complex I activity in the fibroblast cell line from subject 1.

**Figure 4:**
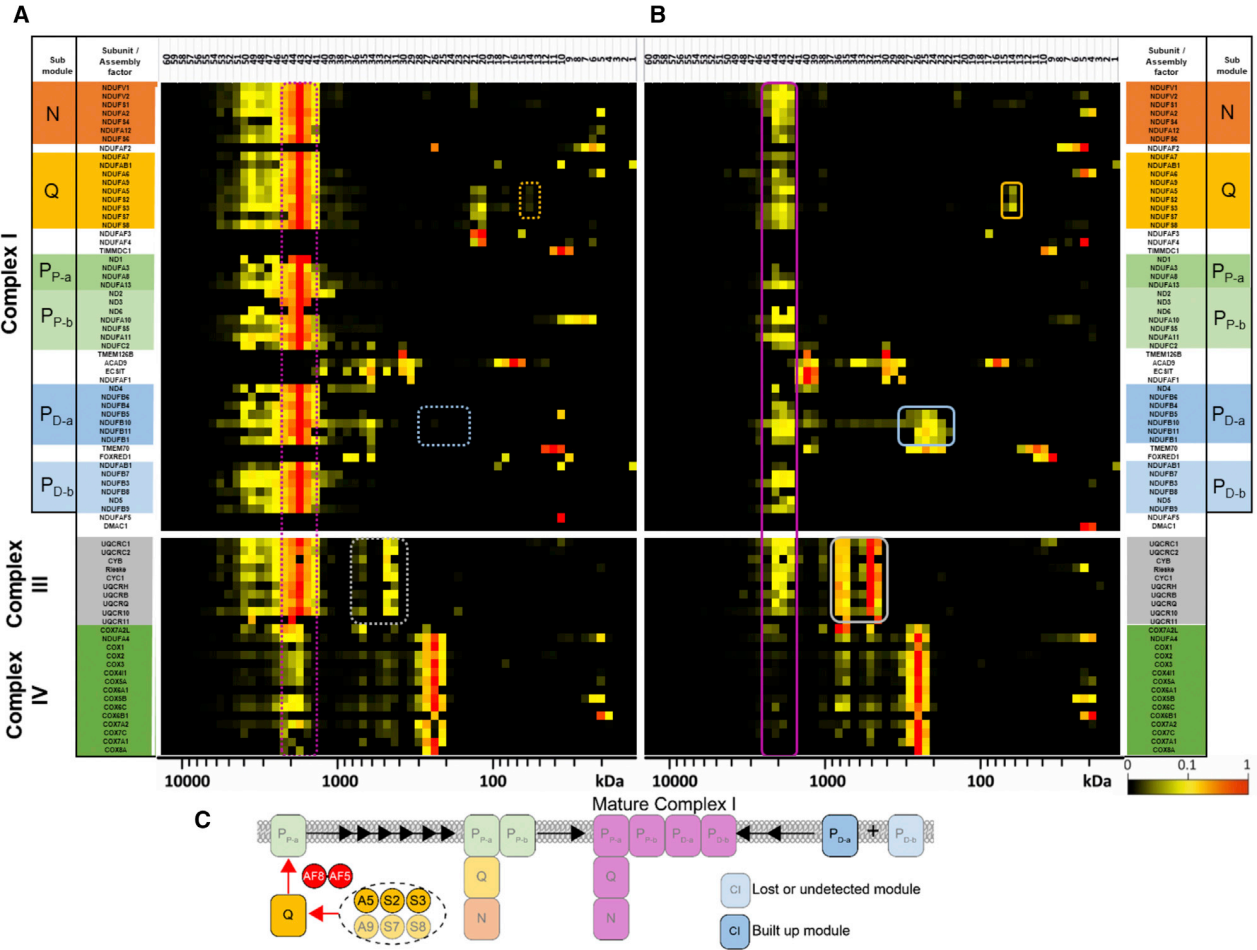
Complexome Profiling of Fibroblasts from Subject 1 Confirms Complex I Assembly Defect Enriched mitochondrial membranes derived from control (A) and subject 1 (B) fibroblasts were solubilized with digitonin, separated on native gradient gels, cut into 60 pieces and digested with trypsin. Peptides were analyzed by quantitative mass spectrometry. Intensity-based absolute quantification (IBAQ) values of subject set were normalized to the sum of all values from control dataset. For visualization in a heatmap, values were normalized to maximum appearance between both samples. There is a reduction in the functional respirasome (complex I/III_2_/IV) (purple box) in subject fibroblasts and stalled assembly intermediates corresponding to P_D-a_ (blue box) and a partly-assembled Q module (orange box), compared to control fibroblast data. There is also an increase in “free” complex III (gray box). Complex I subunits are presented according to their ascribed module, N-module (N), Q-module (Q), proximal P-module (P_P-a_ containing ND1, P_P-b_ containing ND2/3/6), distal P-module (P_D-a_ containing ND4, P_D-b_ containing ND5) and assembly factors (A). Color-matched dashed boxes are used to denote the corresponding regions of interest in the control data (Panel A). Subcomplex denotations are according to Guerrero-Castillo and colleagues^28^ C. Complexome data schematic summarizes the consequence of defective NDUFAF8 on complex I assembly. Complex I subcomplexes are color-coded and named consistent with Panels A and B.

A deeper understanding of the impact of subject 1’s *NDUFAF8* variants on respiratory chain complex assembly and the steady-state levels of other mitochondrial proteins was achieved using complexome profiling, a quantitative mass-spectroscopy assay that has previously been informative in the initial characterization of other complex I genes.^24^^,^^25^ Enriched mitochondrial fractions from subject 1 and control fibroblast cell lines were subjected to BN-PAGE, systematic dissection of the polyacrylamide gel, tryptic digestion, and mass spectroscopy (MS) essentially as reported previously.^25^^,^^26^ MS data, protein identification, quantification, and complete interaction profiles of mitochondrial membranes from subject 1 and control fibroblasts together with methodical details were deposited to the ProteomeXchange Consortium^27^ via the PRIDE partner repository with the dataset identifier PXD015749. Protein abundance in each discrete section of the BN-PAGE gel was transformed for representation as a heatmap (Figure 4). These data show a generalized reduction in complex I subunits in subject 1 fibroblasts (Figure 4B) relative to healthy control fibroblasts (Figure 4A); this result is consistent with the results of initial BN-PAGE analyses. A clear reduction in the assembled respirasome (CI-III_2_-IV) is apparent in subject 1 fibroblasts (Figure 4B, purple box), with a concomitant increase in “free” complex III (Figure 4B, gray box). The current model of complex I (CI) assembly involves multiple pathways that converge to form the final mature CI. The Q module nucleates assembly of the P_P-a_ and P_P-b_ modules, while the N, P_D-a_ and P_D-b_ modules assemble separately. These modules then join to form the complex I holoenzyme.^28^^,^^29^ Given that NDUFAF8 is required for NDUFAF5’s stability, we expect subject 1 to experience the same complex I assembly defects found in subjects with NDUFAF5 mutations, namely defects in Q module assembly.^30^ Indeed, absence of NDUFAF8 results in a stalled Q module assembly involving at least three of the Q module subunits (NDUFS2, NDUFS3, and NDUFA5) (Figure 4B, orange box). Stalling at this early assembly stage results in increased turnover of the later modules, as reflected in decreased levels of CI subunits. The defect in Q module assembly also appears to cause stalling of the P_D-a_ module assembly (Figure 4B, blue box), as well as decreased levels of the P_D-b_ module, summarized in the accompanying schematic (Figure 4C).

NDUFAF8 harbors a twin Cx_9_C motif; therefore it is classified as a member of the Cx_9_C protein family. Most of these proteins exist in the mitochondrial intermembrane space (IMS), including three IMS-facing subunits of complex I (NDUFS5, NDUFB7, and NDUFA8) and one IMS-facing subunit of complex IV (COX6B).^31^ The CHCHD4/ALR (Mia40/Erv1 in yeast) system oxidises the disulfide bonds to create a hairpin-like structure in the IMS. In yeast, the mitochondrial ribosome protein Mrp10p is known to be reduced by this system before import into the matrix.^32^ While it has not been experimentally proven whether NDUFAF8 localizes to the IMS^33^ or matrix,^34^ we presume co-localization of NDUFAF8 to the matrix by virtue of its association with NDUFAF5.^12^^,^^30^^,^^34^

Subjects 1 and 2 both harbor bi-allelic null mutations that are predicted to abolish the NDUFAF8 protein, but subject 3 was found to harbor a homozygous missense variant. The c.165C>G (p.Phe55Leu) substitution involves an invariant amino acid, suggestive of functional importance, but without additional tissue for functional validation, we can only speculate on the mechanism of pathogenicity. The Phe55 residue is situated within a helical domain that forms one side of the predicted hairpin structure of NDUFAF8 (Figure S1A). Based upon TRIAP1, another member of the Cx9C family, the Phe55 residue is predicted to be critical for protein-protein interaction^35^ (Figure S1B), and it could represent a key residue for the interaction between NDUFAF5 and NDUFAF8.^32^ The Phe55 residue could also be critical for protein stability; this possibility is exemplified by a pathogenic variant involving another member of the Cx_9_C family, COA6, in which a pathogenic mutation involving the conserved aromatic Trp59 residue was shown to cause COA6 protein instability.^36^ A screen of variants reported in GnomAD shows that non-synonymous variation involving *NDUFAF8* is rare, particularly involving the conserved aromatic residues; given the small size of NDUFAF8 (74aa, RefSeq: NP_001079990.1), the prevalence of sequence variants was undertaken for all aromatic residues—Trp8, Phe18, Tyr32, Phe50, Phe55, and Phe62. In fact, there are no non-synonymous variants affecting the Trp8, Phe18, Tyr32 or Phe62 residues according to gnomAD v2.1.^37^ Only one heterozygous variant is recorded for the Phe50 and Phe55 residues -c.150C>A p.Phe50Leu (MAF = 3.18x10^−5^; 1/31392 alleles) and c.164T>C p.Phe55Ser (MAF = 7.46x10^−6^, 1/134102 alleles). Although the precise function of NDUFAF8 remains to be elucidated, it is clear that its aromatic residues must be fundamentally important, and this is consistent with subject 3′s rapidly progressive disease course.

It is interesting that subjects 1 and 2 were both reported to have optic nerve involvement, with optic atrophy and a small optic nerve, respectively. Optic atrophy and/or neuropathy is often the predominant feature associated with either *OPA1* mutations or Leber Hereditary Optic Neuropathy (LHON, MIM: 500001), and this is often due to one of three mtDNA point mutations. Each common LHON mutation affects a complex I subunit, m.3460G>A, p.Ala52Thr (ND1); m.11778G>A, p.Arg340His (ND4); or m.14484T>C, p.Met64Val (ND6) (all variants refer to RefSeq: NC_012920.1). Affected individuals typically present in adulthood, although early onset has been reported.^38^ Recessive pathogenic mutations involving *NDUFAF5*^39^ have also been reported in affected individuals with prominent optic nerve involvement. It is widely accepted that the optic nerve is particularly susceptible to complex I dysfunction,^40^ but the link between genotype and phenotype—like many aspects of mitochondrial pathology—remains to be elucidated.

In summary, we describe three young boys whose histories, examinations, and cranial MRI findings supported a clinical diagnosis of Leigh syndrome and who were subsequently found to harbor bi-allelic pathogenic variants in *NDUFAF8*, which encodes a recently identified mitochondrial complex I assembly factor. Through lentiviral rescue of the biochemical complex I deficiency, we unequivocally demonstrate that defective NDUFAF8 is the cause of disease in the subject for whom biological tissues were available. The presence of a recurrent intronic variant in two subjects highlights the importance of thorough interrogation of available intronic sequence data, particularly in cases where a single heterozygous ACMG class 5 variant has been identified. We would recommend that when targeted genetic analysis is undertaken, either by capture or selective analysis of unbiased NGS datasets, sequencing analysis of NDUFAF8 should be undertaken for children with suspected mitochondrial disease, particularly those with optic involvement and/or Leigh syndrome.

## Declaration of Interests

The authors declare no competing interests.

## Accession Numbers

The accession numbers for variants NM_001086521.1:c.1A>G, c.45_52dup, c.195+271C>T, and c.165C>G are ClinVar: SCV000995081, SCV000995082, SCV000995083 and SCV000995084, respectively.

## Web Resources

Addgene, http://www.addgene.org/85132
AlignGVGD, http://agvgd.hci.utah.edu/agvgd_input.php
CADD, https://cadd.gs.washington.edu/
ClinVar, https://www.ncbi.nlm.nih.gov/clinvar/
Ensembl, www.ensembl.org/
ExAC Browser, http://exac.broadinstitute.org/
GenBank, https://www.ncbi.nlm.nih.gov/genbank/
gnomAD, http://gnomad.broadinstitute.org/
OMIM, https://www.omim.org/
PolyPhen-2, http://genetics.bwh.harvard.edu/pph2/
SIFT, http://sift.bii.a-star.edu.sg/
UCSC Genome Browser, https://genome.ucsc.edu/
UniProt, https://www.uniprot.org/

## Supplemental Data

Document S1. Figures S1–S6 and Supplemental References

Document S2. Article plus Supplemental Information
