# Supplementary material for: Pathogenic Bi-allelic Mutations in NDUFAF8 Cause Leigh Syndrome with an Isolated Complex I Deficiency
Source: Am J Hum Genet. 2019 Dec 19;106(1):92–101. doi: 10.1016/j.ajhg.2019.12.001 (PMC7042492; doi:10.1016/j.ajhg.2019.12.001)
Supplement: Document S1. Figures S1–S6 and Supplemental References [file mmc1.pdf]

The American Journal of Human Genetics, Volume *106*

## Supplemental Data

# Pathogenic Bi-allelic Mutations in *NDUFAF8* Cause Leigh Syndrome with an Isolated Complex I Deficiency

**Charlotte L. Alston, Mike T. Veling, Juliana Heidler, Lucie S. Taylor, Joseph T. Alaimo, Andrew Y. Sung, Langping He, Sila Hopton, Alexander Broomfield, Julija Pavaine, Jullianne Diaz, Eyby Leon, Philipp Wolf, Robert McFarland, Holger Prokisch, Saskia B. Wortmann, Penelope E. Bonnen, Ilka Wittig, David J. Pagliarini, and Robert W. Taylor**

## Supplemental Figures

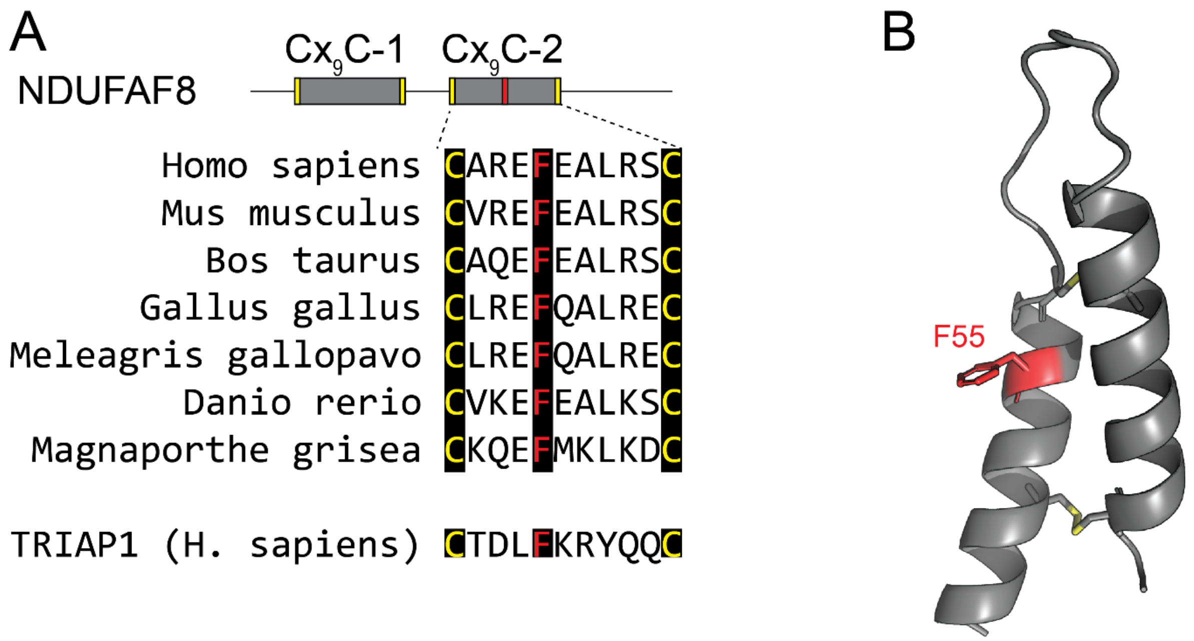


**Supplemental Figure S1. Conservation of the Phe55 residue and predicted tertiary structure of NDUFAF8.**

**A** Alignment of the second $Cx_9C$ motif across several species with NDUFAF8 according to CLIME [1]. Human TRIAP1 is also included in the alignment as its crystal structure was used to model NDUFAF8 (**B**). **B** SWISS MODEL prediction of the tertiary structure of NDUFAF8 based on the crystal structure of TRIAP1 [2] demonstrating the disulfide bonds arising from the twin $Cx_9C$ motifs. The p.Phe55 variant that is mutated in Subject 3 is labelled (F55) and is situated within the $Cx_9C$-derived hairpin.

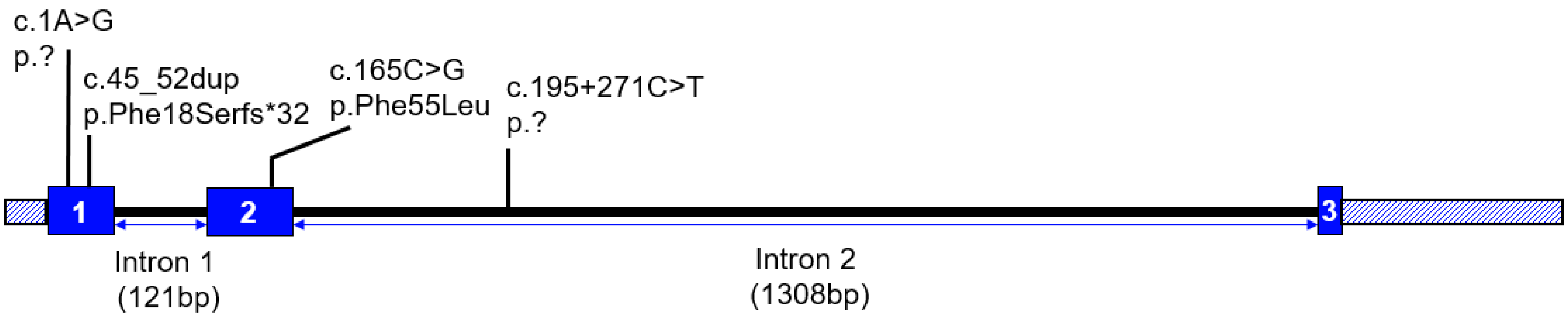


**Figure S2: Pathogenic *NDUFAF8* variants and gene structure.**

*NDUFAF8* is a small gene (<3kb genomic DNA) with just three exons. Identified gene variants are mapped onto the gene schematic; the small size of the introns facilitated identification of the intronic c.195+271C>T variant in the whole exome sequencing data for Subject 2.

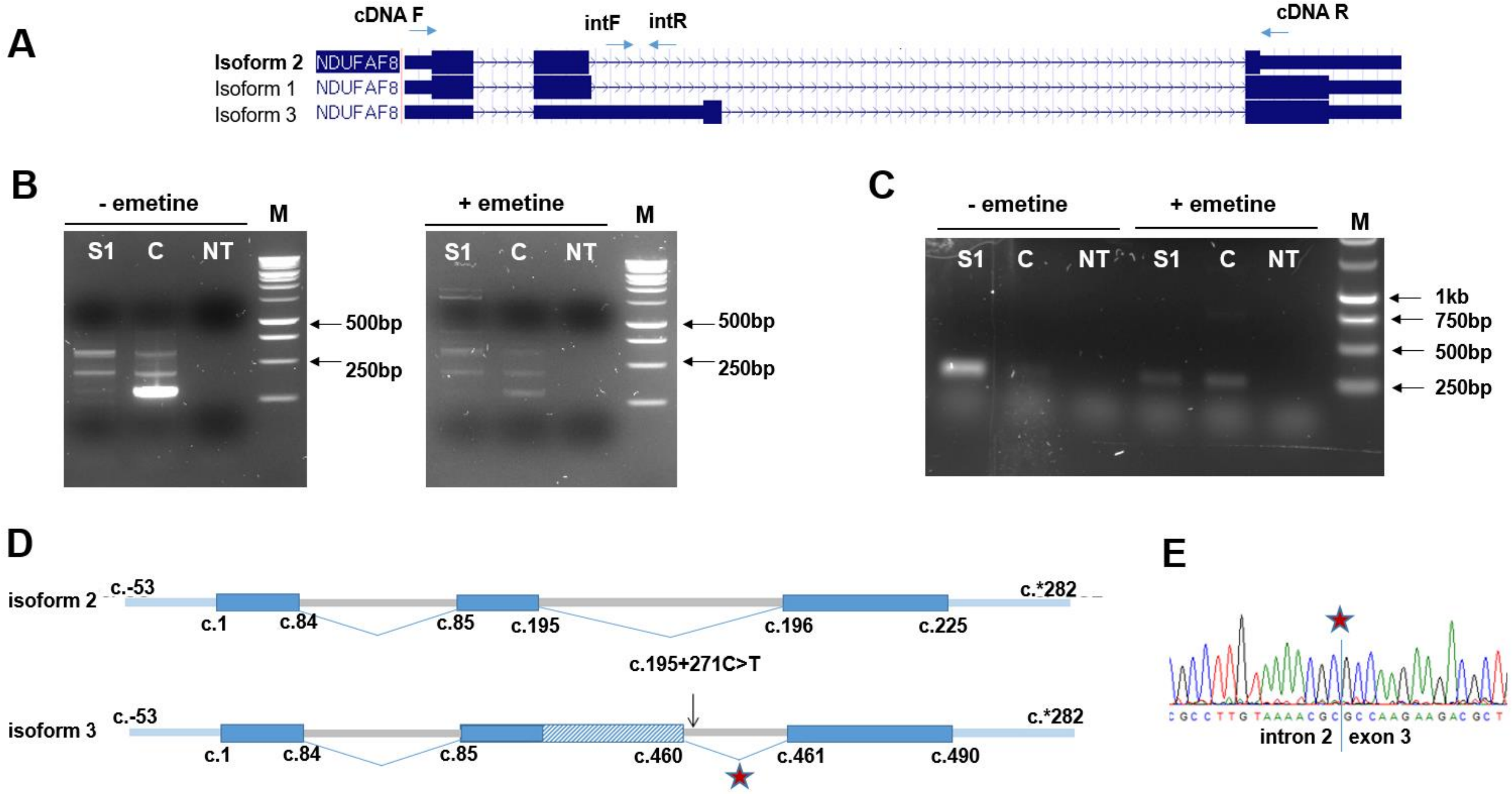


**Figure S3: cDNA analysis of Subject 1 fibroblasts provides evidence of aberrant splicing.**

**A.** Three isoforms of NDUFAF8 exist; the functional isoform being isoform 2. Primers used for the NDUFAF8 cDNA amplification are denoted cDNA F/R (full length NDUFAF8 cDNA molecule, with primers common to all three transcripts) and a sequencing intF/R primer, specific to

isoform 3. **B.** Amplification products of Subject 1 (S1) and Control (C) fibroblast-derived cDNA samples using NDUFAF8 cDNAF/R primers, with or without overnight exposure to emetine; nt=no template; M=Promega 1kb ladder. A clear reduction in the smallest (~150bp) amplicon is observed in Subject 1 untreated fibroblasts compared to the control. Additionally, high molecular weight amplicons are present in Subject 1's emetine-treated fibroblasts, compared to the control, suggestive of aberrantly spliced transcripts that are anticipated to be subject to nonsense mediated decay *in vivo*. **C.** Amplification of Subject 1 and control fibroblast-derived cDNA using a forward primer specific to NDUFAF8 isoform 3 is supportive of considerably higher levels compared to control; the presence of NDUFAF8 isoform 3 at apparently similar levels in emetine treated cells support the predominant turnover of this isoform *in vivo.* **D.** Retention of intronic sequence distinguishes isoforms 2 and 3, a star denotes the junction, visualised in the sequencing chromatogram (**E**).

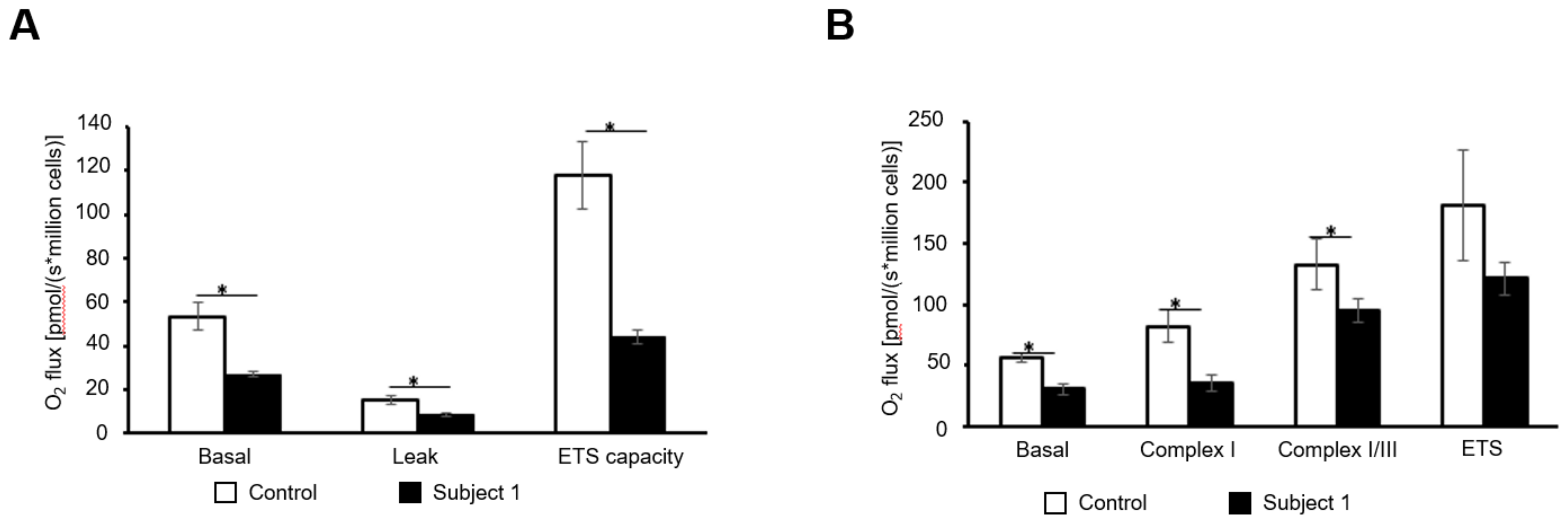


**Supplemental Figure S4: Mutation of NDUFAF8 causes dramatic defects on cell respiration and complex I.**

**A.** High-resolution respirometry of patient-derived fibroblasts (black bars) compared to control fibroblasts (white bars). Basal- oxygen consumption rate of intact cells in growth medium, Leak- oxygen consumption rate upon ATP synthase inhibition with Oligomycin, ETS capacity- maximal uncoupled respiration. Data are mean ± SD from 3 experiments. **p<0.01; values are mean±SD. **B.** High-resolution respirometry of patient-derived fibroblasts (black bars) compared to control fibroblasts (white bars). After measurement of basal respiration cells were permeabilized with digitonin and mitochondrial respiratory rates are measured for complex I (CI) and complex I+II (CI+II) OXPHOS capacity followed by the measurement of maximal uncoupled respiration after stepwise titration of FCCP (ETS). Data are mean ± SD from 3 experiments.*p<0.05, **p<0.01; values are mean±SD

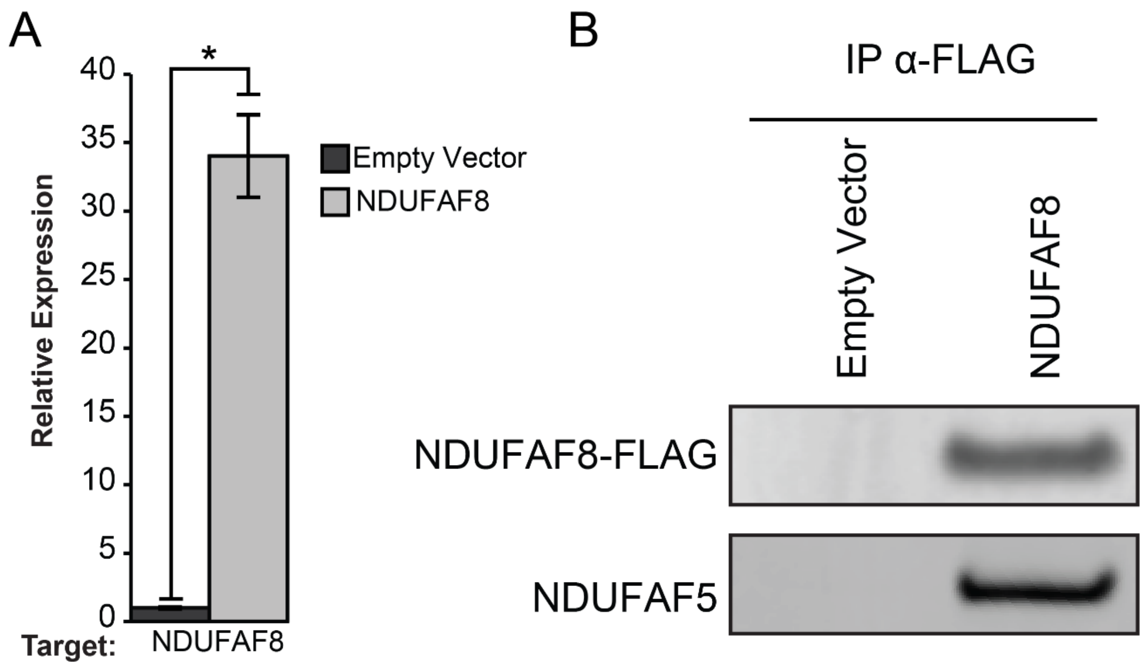


**Supplemental Figure S5. NDUFAF8-FLAG is being expressed in the EF1α based rescue cell line.**

**A**, qPCR of NDUFAF8 levels using primers targeting both endogenous NDUFAF8 as well as the NDUFAF8-FLAG overexpression construct. Error bars are at ±1 standard deviation, data shown are from three technical replicates.

**B,** α-FLAG IP from transformed cell lines Western blotted for FLAG or NDUFAF5.

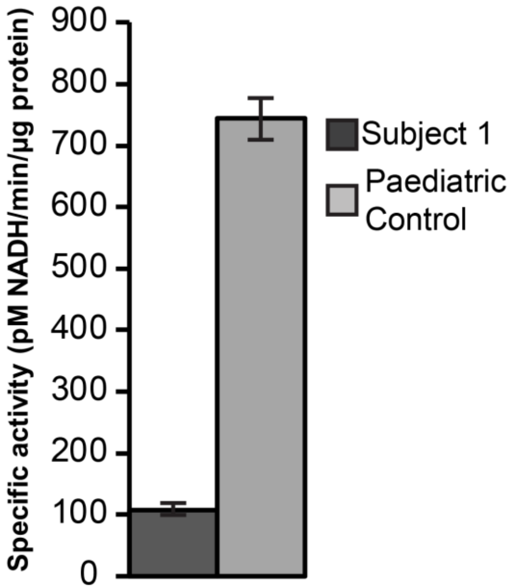


**Supplemental Figure S6. Complex I activity in primary fibroblasts from Subject 1 is impaired.** Spectrophotometric analysis using a colorimetric complex I activity assay (ab109721, Life Technologies) demonstrates a marked complex I deficiency in the primary fibroblast cell line from Subject 1 compared to a paediatric control. Error bars are at ±1 standard deviation, data shown are from three technical replicates.

**Supplemental references**

1. Li Y, Calvo SE, Gutman R, Liu JS, Mootha VK. Expansion of biological pathways based on evolutionary inference. *Cell.* 2014;158(1):213-25.

2. Miliara X, Garnett JA, Tatsuta T, Abid Ali F, Baldie H, Pérez-Dorado I, Simpson P, Yague E, Langer T, Matthews S. Structural insight into the TRIAP1/PRELI-like domain family of mitochondrial phospholipid transfer complexes. *EMBO Rep.* 2015;16(7):824-35.
